# Changes in Resting-State Functional Connectivity of Cerebellum in Amnestic Mild Cognitive Impairment and Alzheimer’s Disease: A Case-Control Study

**DOI:** 10.3389/fnsys.2021.596221

**Published:** 2021-03-10

**Authors:** Zhi Zhou, Rui Zhu, Wen Shao, Shu-juan Zhang, Lei Wang, Xue-jiao Ding, Dan-tao Peng

**Affiliations:** ^1^Department of Neurology, China-Japan Friendship Hospital, Beijing, China; ^2^Department of Neurology, Beijing Geriatric Hospital, Beijing, China

**Keywords:** Alzheimer’s disease, amnestic mild cognitive impairment, cerebellum, functional connectivity, resting state fMRI

## Abstract

This case-control study is aimed to investigate the correlation of altered functional connectivity (FC) in cerebellum with cognitive impairment in amnestic mild cognitive impairment (aMCI) and Alzheimer’s disease (AD). The morphometric and resting-state FC MRI analysis including 46 participants with AD, 32 with aMCI and 42 age-matched normal controls (NCs) were conducted. We compared the cerebellar gray matter volume and cerebellar FC with cerebral cortical regions among three groups. To investigate the relationship of cerebellar FC with cognition, we measure the correlation of significant altered FC and individual cognitive domain. No significant morphometric differences of cerebellum was observed across three groups. The patients with AD had weaker cerebral cortical FCs in bilateral Crus I and left VIIb compared to NCs, and in bilateral Crus I compared to patients with aMCI. For patients with aMCI, the weaker FC were found between right Crus I, left VIIb and cerebral cortical regions compared to NCs. The strength of left cerebellar FC positively correlated with specific cognitive subdomains, including memory, executive function, visuospatial function, and global cognition in AD and aMCI. These findings demonstrated the alteration of cerebellar FC with cerebral cortical regions, and the correlation of cerebellar FC and cognitive impairment in AD and aMCI.

## Introduction

Alzheimer’s disease (AD) is the most common form of dementia, leading to a heavy burden on patients, family and society. The hallmark of the pathology of AD is the deposition of amyloid-β (Aβ) in the cerebrum. The cerebellum has been recognized as essential only for the motor control and being free of Aβ deposition (Guo et al., 2016; Jacobs et al., 2018). Therefore, for a long time, the cerebellum was considered as a “standby” in AD, and was widely used as reference region for the calculation of standardized uptake value ratio (SUVR) in molecular imaging studies (Clark et al., 2011).

However, increasing evidences demonstrate that the cerebellum is also associated with the regulation of cognition by way of the cerebrocerebellar circuits (Schmahmann et al., 2019). With the advancement of staining techniques, pathological studies also found the deposition of Aβ, neurofibrillary tangles and increased microglia on cerebellar cortex in AD (Rudzinski et al., 2008; Sepulveda-Falla et al., 2014). Increasing neuroimaging studies reported the cerebellar atrophy and functional alteration in AD (Bai et al., 2011; Wang et al., 2011; Lou et al., 2015; Mascali et al., 2015; Guo et al., 2016; Jacobs et al., 2018; Olivito et al., 2020). However, till now, the studies about the changes of cerebellum in AD patients are still limited, and results are not consistent. This inconsistency may be due to the poor overlap of cerebellar subregions in parcellation by conventional whole-brain methods. Moreover, most previous studies focused on the relation between cerebellum and global cognitive function, the specific cognitive domain correlates with the alteration of cerebellum remains uncertain. More evidence is needed to illustrate the role of cerebellum in AD.

We hypothesized that altered cerebellar volume and functional connectivity (FC) correlated with cognitive dysfunction, and correlated with cognitive impairment in AD continuum. As amnestic mild cognitive impairment (aMCI) has a high incidence of conversion to AD, aMCI also provides a good model to investigate subtle change at the initial stage of the AD continuum (Petersen et al., 2001). Comparing the change of cerebellum among normal controls (NCs), patients with aMCI and AD, could help us to illustrate the processing of change in different cognitive status. With a cerebellum-specific spatially unbiased infratentorial template (SUIT), we performed voxel-based morphometry (VBM) analysis to compare the cerebellar volume between patients across different cognitively populations, including AD, aMCI and NCs. In addition, we used resting-state functional MRI (rs-fMRI) to investigate the FC between cerebellum and cerebral cortical regions, and the relations between altered FC and specific cognitive domains were calculated.

## Materials and Methods

### Study Design and Participants

This is a retrospective case-control study. Participants were recruited in preparation for this study from the memory clinic of China-Japan Friendship Hospital from 2014 to 2019. Participants with structural and rs-fMRI images were enrolled using the inclusion and exclusion criteria below. Patients with AD met the diagnostic criteria of probable AD dementia according to the new National Institute on Aging-Alzheimer’s Association criteria of 2011 (McKhann et al., 2011). The inclusion criteria for AD included: (1) significant episodic memory problems reported by the patient, relative or caregiver, which was corroborated by the score of Rey Auditory Verbal Learning Test (AVLT); (2) impaired performance on general cognition test (Mini-Mental State Examination (MMSE) score < 24) and activities of daily living (ADL); (3) medial temporal lobe atrophy on visual atrophy rating scale (Scheltens et al., 1992). Patients with aMCI participants satisfied with the Petersen’s criteria and the National Institute on Aging-Alzheimer’s Association criteria for MCI due to AD (van den Burg and Kingma, 1999; Petersen et al., 2014). The inclusion criteria were as follows: (1) memory complaint; (2) scoring lower than 1.5 standard deviations of the age- and education-adjusted norm on the score of AVLT; (3) normal performance on general cognition test (MMSE score ≥ 24) and ADL. The NCs included family members of patients, who did not have cognitive complaints or significant decline on the neuropsychological testing, and with MMSE score ≥ 24. The NCs were matched with AD and aMCI participants in gender and age.

Exclusion criteria for all participants included: (1) current or previous history of significant neurological disorder that could cause cognitive decline, including stroke, epilepsy, head trauma, intracranial mass or normal pressure hydrocephalus; (2) history of addictions or other psychiatric disorders, including schizophrenia, bipolar disorder or depression; (3) other severe medical problems, including chronic heart failure and chronic respiratory insufficiency; (4) left handed.

### Clinical and Neuropsychological Assessment

All participants underwent neurological evaluation and comprehensive neuropsychological assessment. The neuropsychological assessments included general cognitive status and a series of detailed cognitive tests for specific cognitive domains, including memory, language, executive function, attention and visuospatial function (Supplementary Material 1 provide the details). The *z* score of each cognitive domain and the composite cognitive *z* score (average of the five individual cognitive domains) were computed based on normative data from 114 healthy control participants with similar age, education, and gender distribution (age: 69.8 ± 6.4; Male/Female: 47/67, education 14.8 ± 2.8 years, no history of neurological or psychiatric illness) in our center. Neuropsychiatric symptoms and functional impairment were assessed by caregiver-based questionnaires: Neuropsychiatric Inventory (NPI) and ADL, respectively (Cummings, 1997). The APOE genotype was determined from genotyping of isolated DNA from blood. The participants who had at least 1 APOE ε4 allele were considered as APOE ε4 carriers.

### MRI Data Acquisition and Preprocessing

The rs-fMRI images and T1-weighted MRI images were acquired using a 3.0 T MR imaging system (GE Healthcare, Discovery MR750, Milwaukee, WI, United States) in the Radiology Department of China-Japan Friendship Hospital. The parameters of sagittal three-dimensional T1-weighted images with fast spoiled gradient-echo sequences (FSPGR) were as follows: echo time (TE) = 3.0 ms, repetition time (TR) = 6.9 ms, slice thickness = 1.0 mm, FOV = 256 mm × 256 mm, acquisition matrix = 256 × 256, and flip angle = 12°. The parameters of axial resting-state data were as follows: TE = 30 ms, TR = 2,000 ms, slice thickness = 3.0 mm, 33 slices, field of view (FOV) = 240 mm × 240 mm, in plane matrix = 64 × 64, flip angle = 90°, and 240 phases.

Structural three-dimensional (3-D) T1 images were first processed using the SUIT toolbox^1^ implemented in the Statistical Parametric Mapping software version 12 (SPM12)^2^ toolbox (Diedrichsen et al., 2009). Each cerebellum was separated by a Bayesian algorithm into gray matter (GM) and white matter (WM), normalized to the Montreal Neurological Institute (MNI) space using the high-resolution probability template in SUIT. The intensity of each voxel was modulated to conserve the regional differences in the total amount of GM. All the images were smoothed with a 4-mm full-width at half-maximum (FWHM) Gaussian kernel.

The rs-fMRIs were preprocessed with the Data Processing Assistant for Resting-State fMRI (DPARSF) and the Resting-State fMRI Data Analysis Toolkit (REST). First, the first 10 volumes were discarded for the signal equilibrium and adaptation of subjects to the scanning noise. The remaining 230 volumes were corrected for timing difference and realigned to the first volume to correct for possible movement. The frame-wise displacement (FD) (Jenkinson) was calculated to evaluate the mismatch of volume-to-volume superimposed head position. The mean FD for the all the participants were 0.21 ± 0.14 mm. The data of 6 subjects (4 AD, and 2 NC) were excluded in this step due to excessive head motion (greater than 2.5 mm, greater than 2.5° angular rotation or mean FD > mean FD + 2SD). After removing the 6 subjects, the FD showed no significant different among the different groups (AD: 0.19 ± 0.09 mm, aMCI: 0.18 ± 012 mm; NC: 0.19 ± 0.09 mm, *F*-value estimated by one-way ANOVA was 0.45, *p* = 0.64). To normalize the resting images, the T1 images were registered to their corresponding functional images and were then segmented into GM, WM, and cerebrospinal fluid tissue (CSF) probabilistic maps using a unified segmentation algorithm. Second, a GM population template was derived from the whole image data set with the DARTEL technique. Third, non-linear warping of the segmented images was then performed to match the MNI space DARTEL template. Spatial smoothing was then performed with an isotropic 4-mm FWHM Gaussian kernel. Next, linear detrending and temporal band-pass filtering (0.01–0.1 Hz) were applied to remove low-frequency drifts and high-frequency noise. Finally, the nuisance variables (including 6 head motion parameters and their derivatives, the WM and CSF signal, and the linear term) were regressed out.

### Seed ROIs

**FIGURE 1:**
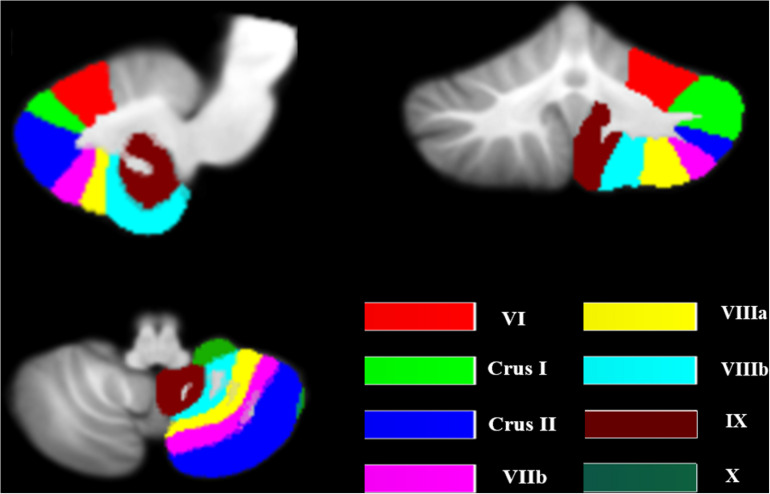
The seeds of the cerebellum. The image was transformed into the space of the SUIT atlas and was overlapped by the seeds. The different colors show the lobular parcellation.

Previous studies revealed that the posterior lobe (VI, VIIb, VIII), ansiform lobe (Crus I, Crus II), and flocculonodular lobe (IX, X) of cerebellum are especially associated with cognition (Barton and Harvey, 2000; Whiting and Barton, 2003; Prevosto et al., 2010; Stoodley and Schmahmann, 2010). Therefore, these lobules were included as seed ROIs. The masks of these ROIs were extracted from the probabilistic cerebellar atlas used in SUIT (Diedrichsen et al., 2009; Figure 1). For each participant, the voxel of each seed was extracted to obtain the average seed point time series. A correlation coefficient map for each seed was produced by correlating the coefficients between the reference time series and the time series from all other brain voxels, which was then transformed to Fisher *z*-values.

### Statistical Analysis

Data were analyzed using SPSS 22.0 (IBM Corp., Chicago, IL, United States). Demographic and clinical variables were checked for normality of distribution using Kolmogorov–Smirnov tests. Variables revealing normal distribution were compared across groups via ANOVA followed by Bonferroni *post hoc* tests if ANOVA was significant (*p* < 0.05). Group comparisons of NPI and ADL between AD and aMCI were performed using Student’s *t* test. Gender and ApoE4 status data were analyzed using a Chi-square test. *p* < 0.05 was regarded as significant.

The resulting images were subsequently entered into a VBM analysis to perform the one-way ANOVAs to identify differences among groups in the cerebellum. Age, gender, and total intracranial volume were included as nuisance covariates. Statistical analyses were performed on smoothed GM maps within the framework of the general linear model. A one-way ANOVA model was used for assessing between group differences in regional GM cerebellar volumes. Age, sex, and years of education were included as covariates in our analysis.

ANOVA analysis was used to compare the whole-brain FC of each seed among the three different groups. The age, gender, education level, head movement parameters, and total intracranial volume were used as covariates. False discovery rate (FDR) correction was performed with a threshold of 0.05. The *z*-values of FC were extracted to perform the *post hoc t* test in order to identify the inter-group differences between AD and NC, aMCI and NC, and aMCI and AD. Bonferroni correction was performed to adjust for the multiple testing, with a *p* value of <0.0167 (0.05/3) considered statistically significant for these comparisons.

Due to the small sample size of individual group, we combined the patients with aMCI and AD together to investigate the association between all the significant different FC and the five individual cognitive subdomain. When combined aMCI and AD groups together, the *z* scores of each cognitive subdomain did not present a normal distribution (Shapiro-Wilk test, *p* < 0.05), therefore, the Spearman’s correlation was used. The Bonferroni correction was used for multiple comparisons correction with *p* < 0.01 (0.05/5) was considered significant.

### Confirmatory Analysis Based on the ADNI Dataset

The Alzheimer’s disease Neuroimaging Initiative (ADNI) database^3^ was used to verify the results obtained from our data. Participants who with availability of T1-weighted MRI and rs-fMRI images were selected in the confirmatory study. The T1-weighted MRI images were performed as follows TE = 3.13 ms, TR = 6.77 ms, voxel size = 1 mm isotropic, FOV = 256 mm × 256 mm, acquisition matrix = 256 × 256. The parameters of rs-fMRI data were as follows: TE = 30 ms, TR = 3,000 ms, 48 slices, voxel size = 3.3125 mm isotropic, FOV = 256 mm × 256 mm, and 140 phases. The full details of imaging data^4^ can be found on the ADNI web site. 189 participants were recruited, however, 11 participants were excluded because of the poor quality of rs-fMRI data or excessive head motion. Finally, subjects with AD (*n* = 54), MCI (*n* = 57) and NC (*n* = 67) were selected. Details of the diagnostic criteria were from the ADNI web site^5^.

## Results

### Demographic and Neuropsychological Results

**FIGURE 2:**
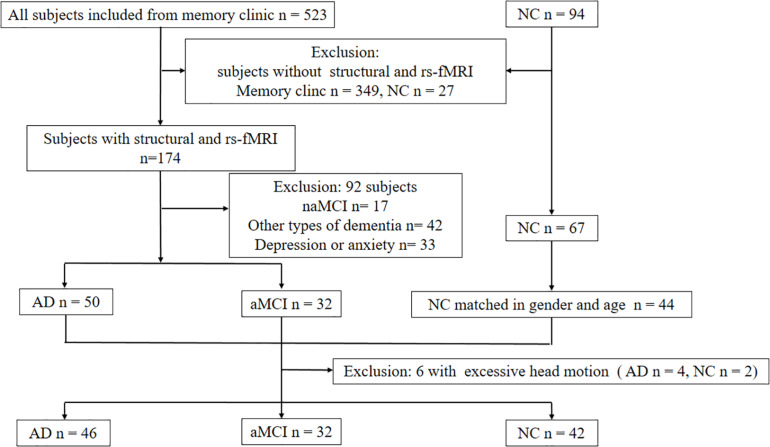
Diagram showing the number and flow of subjects in this study. Abbreviations: NC: normal control; aMCI: amnestic mild cognitive impairment; naMCI: non-amnestic mild cognitive impairment; rs-fMRI: resting-state functional MRI.

From June 2014 to June 2019, we recruited 46 subjects with AD, 32 aMCI, and 42 NCs with the aforementioned procedures (Figure 2). Table 1 shows the clinical and neuropsychological data. No significant differences in age, gender and education level. Regarding the cognitive performance, the AD group had significantly lower scores than both the aMCI and NC groups on general cognitive test and all the cognitive subdomains except attention. aMCI subjects had significantly lower score on memory, language and executive function than NC subjects. The patients with AD scored significantly higher score on the ADL and NPI compared to patients with aMCI.

**TABLE 1:** Demographic and neuropsychological data.

	Normal controls	Amnestic MCI	Alzheimer’s disease	*p* (ANOVA)
***N***	42	32	46	
Age, years	69.86 ± 6.66	70.84 ± 7.54	73.17 ± 7.09	0.083
Gender (Male,%)^4^	18 (42.86%)	16 (50.0%)	19 (41.30%)	0.732
Education, years	14.35 ± 3.05	13.68 ± 3.43	12.74 ± 4.32	0.153
MMSE	29.29 ± 0.86	26.16 ± 1.65	18.52 ± 3.48	<0.001^1, 2, 3^
MoCA	27.09 ± 1.48	21.72 ± 2.87	14.02 ± 4.26	<0.001^1, 2, 3^
NPI^5^	-	6.41 ± 5.21	12.17 ± 11.99	0.013
ADL^5^	-	23.59 ± 3.43	34.02 ± 8.37	<0.001
APOE ε4 carrier (n,%)^4^	11 (23.81%)	18 (59.38%)	29 (63.04%)	<0.001
Composite cognitive z score	0.04 ± 0.44	−1.03 ± 0.73	−2.02 ± 1.01	<0.001^1, 2, 3^
*z*-memory	0.12 ± 0.71	−2.10 ± 0.49	−2.70 ± 0.83	<0.001^1, 2, 3^
*z*-language	0.26 ± 0.62	−1.29 ± 1.28	−2.00 ± 1.13	<0.001^1, 2, 3^
*z*-executive function	−0.05 ± 0.76	−0.62 ± 1.31	−1.16 ± 1.01	<0.001^1,3^
*z*-attention	−0.07 ± 0.95	−0.30 ± 1.32	−0.63 ± 1.03	0.053
*z*-visuospatial	−0.10 ± 0.86	−0.85 ± 1.12	−3.21 ± 2.43	<0.001^1, 3^

*MMSE, mini-mental status examination; MoCA, Montreal Cognitive Assessment; NPI, Neuropsychiatric Inventory; ADL, Activity of Daily Living.*

*(1). *Post hoc* analysis showed significant group differences between NC and AD.*

*(2). *Post hoc* analysis showed significant group differences between NC and aMCI.*

*(3). *Post hoc* analysis showed significant group differences between aMCI and AD.*

*(4). Values were mean ± standard deviation. Comparisons using Chi-square test.*

*(5). Values were mean ± standard deviation (sd). Comparisons using Student’s *t* test.*

### Cerebellar Morphometry

No significant group difference in volume of any cerebellar lobular was found among three groups with predefined threshold.

### Seed-Based FC

**FIGURE 3:**
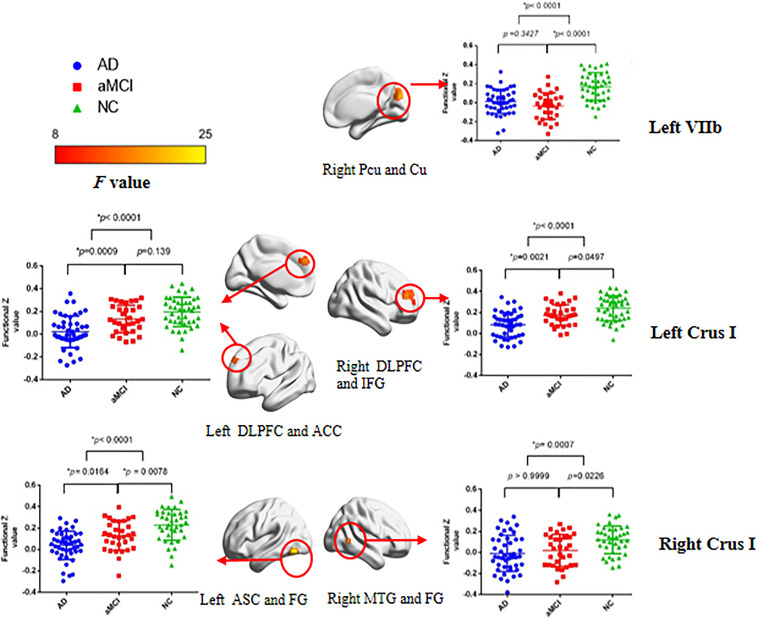
Statistical parametric map and the scatterplot of the significant cerebral cortical clusters among three groups and the *post hoc* analysis between groups. Each row corresponds to a distinct cerebellar lobular seed region of interest. The difference remained significant after Bonferroni correction at *post hoc* analysis. Color bar represents *F* values. BrainNet Viewer (Beijing Normal University, http://www.nitrc.org/projects/bnv/) was used for the visualization of the results. L, left; R, right; Bi, bilateral; PCu, precuneus; Cu, cuneus; DLPFC, dorsolateral prefrontal cortex; IFG, inferior frontal gyrus; ACC, anterior cingulate cortex; ASC, associative visual cortex; MTG, middle temporal gyrus; FG, fusiform gyrus.

Table 2 and Figure 3 illustrate the significant difference clusters in the FC for each seed among three groups. The patients with AD had weaker cerebral cortical FCs in bilateral Crus I, left VIIb compared to NCs. The weakened cerebellar FCs with visual cortex [Brodmann area, (BA) 18, 19), including precuneus and cuneus, were found in left VIIb and IX in AD. The left Crus I in AD had weaker correlations with dorsal lateral prefrontal cortex (DLPFC), inferior frontal gyrus (IFG), and anterior cingulate cortex (ACC) (BA 9, 32, 45, 46). The weakened correlations with associative visual cortex (ASC), fusiform gyrus (FG) and middle temporal gyrus (MTG) (BA19, 21, 37) were found in right Crus I. Among cerebral cortical FCs mentioned above, the weakened FCs in bilateral Crus I still showed significant compared to patients with aMCI. For patients with aMCI, the right Crus I, and Left VIIb and IX was found with significantly weaker cerebral cortical FC compared to NCs.

**TABLE 2:** Brain regions showing significant differences during one-way ANOVA on *z* value of functional connectivity maps of NC, aMCI, and AD groups.

Seed	Cluster voxels	Brain regions	Laterality	BA	MNI coordinate			Maxi-mum *F*
					*x*	*y*	*z*	
Left VIIb	13	Visual cortex (Pcu and Cu)	Right	18, 19	9	−84	27	18.24
Left Crus I	44	DLPFC and IFG	Right	45, 46	42	42	18	17.29
	19	DLPFC and ACC	Left	9, 32	−6	48	33	17.23
Right Crus I	23	MTG and FG	Right	21, 37	−45	−69	−12	25.18
	15	ASC and FG	Left	19, 37	60	−57	6	24.62

*BA, Brodamann area; PCu, precuneus; Cu, cuneus; DLPFC, dorsolateral prefrontal cortex; IFG, inferior frontal gyrus; ACC, anterior cingulate cortex; MTG, middle temporal gyrus; FG, fusiform gyrus; ASC, associative visual cortex.*

Cerebral cortical FC in other seeds of cerebellum was not significantly different among three groups.

### Cognitive Correlations of Cerebellar FC

**FIGURE 4:**
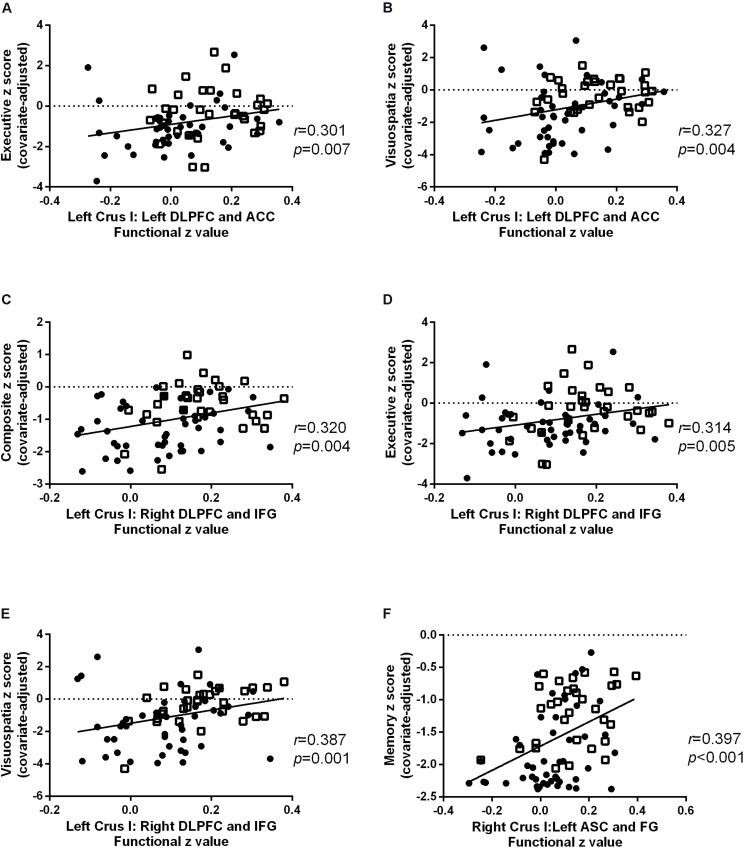
Scatter plots for the significant cognitive-functional connectivity (FC) correlations in Alzheimer’s disease (AD) (circles) and amnestic mild cognitive impairment (aMCI) (squares). The strength of FC with left dorsolateral prefrontal cortex (DLPFC) and inferior frontal gyrus (IFG) positively correlated with **(A)** executive and **(B)** visuospatial function. The FC of left Crus I and correlated with **(C)** global cognition, **(D)** executive function, and **(E)** visuospatial function. The FC of right Crus I with left associative visual cortex (ASC) and fusiform gyrus (FG) positively correlated with memory **(F)**.

Cognitive correlates of the FC findings in patients with AD and aMCI were investigated for all reported significant cerebellar-cerebral cortical FCs after controlling for age, gender and education (Table 3 and Figure 4). For left Crus I, the strength of FC with left DLPFC and ACC (BA 9, 32), positively correlated with executive function and visuospatial function (Figures 4A,B). The strength of FC between left Crus I and right DLPFC and IFG (BA 45, 46) correlated with global cognition, executive function and visuospatial function (Figures 4C-E). The FC of right Crus I with left ASC and FG correlated with memory (Figure 4F). No significant correlation with other cognitive subdomain was found. In the cerebral cortical FC with Left VII, IX, no significant correlation was found with individual cognitive domain and global cognition. The correlation for aMCI and AD subgroup is detailed in Supplementary Table 2.

**TABLE 3:** Spearman Correlation between cognition and cerebellar FC.

BA			Composite *z* score	Memory *z* score	Language *z* score	Executive *z* score	Attention *z* score	Visuospatial *z* score
Left VIIb	Right BA 18, 19	***r***	0.018	0.010	−0.085	0.057	−0.070	0.062
		***p***	0.876	0.930	0.457	0.619	0.548	0.594
Left Crus I	Left BA 9,32	***r***	0.270	0.159	0.161	0.301*	0.094	0.327*
		***p***	0.017	0.164	0.159	**0.007**	0.415	**0.004**
	Right BA 45, 46	***r***	0.320*	0.191	0.252	0.314*	0.054	0.387*
		***p***	**0.004**	0.093	0.026	**0.005**	0.644	**0.001**
Right Crus I	Left BA 19, 37	***r***	0.204	0.397*	0.272	0.241	0.095	−0.030
		***p***	0.073	**0.000**	0.016	0.034	0.413	0.795
	Right BA 21, 37	***r***	0.138	0.233	0.143	0.116	0.144	0.030
		***p***	0.230	0.040	0.213	0.313	0.213	0.794

BA, Brodamann area. Bold and * means *p* < 0.05.

### Results of the Confirmatory Analysis on the ADNI Dataset

Results of the ADNI data showed similarities and differences with those obtained from our sample.

In the VBM analysis, compared with the NC group, the AD and aMCI groups had significant gray matter volume reduction in the left Crus I/II, and there is no difference between the AD and aMCI group (NC > AD: *p* < 0.001; NC > aMCI: *p* < 0.001; aMCI > AD: *p* = 0.059). Supplementary Table 3 and Supplementary Figure 1 show the details.

The seed-based FC analysis based on ADNI database showed that the cortical FC in left IX, left Crus I and bilateral Crus II are involved in aMCI group, with a later involvement of the right X lobe in AD group only. Similar to our sample, the weakened cortical cerebellar FCs involved left DLPFC, FG, bilateral precuneus and cuneus. Details are illustrated in Supplementary Table 4 and Supplementary Figures 2–5.

## Discussion

This case-control study investigated the cerebellar anatomic and functional changes across three different cognitive status, including the NC, aMCI, and AD group. The strength of cerebellar FC with cerebral cortical areas were different among three groups, and it correlated with cognitive function in AD and aMCI. The results from ADNI cohort partially confirmed these findings.

The weakened FC was found between the left VIIb and contralateral precuneus and cuneus in AD. Precuneus is one of the core regions of default mode network (DMN) (Buckner et al., 2008). Aβ accumulation preferentially starts in several of the core regions of the DMN, including the precuneus at the early stage of AD (Palmqvist et al., 2017). From the perspective of clinical symptoms, the DMN has been found to be related to episodic memory in AD (Buckner et al., 2008; Sperling et al., 2009). Failure to detect the correlation with the cognitive performance, especially with the memory, could be due to the restricted range of this dependent variable in our aMCI cohort.

Compared to NC, the aMCI and AD group showed weaker FC of the left Crus I correlated with frontal lobe (bilateral DLPFC, left ACC and right IFG), while right Crus I with occipital and temporal lobule (bilateral FG, left ASC and right MTG). The role of Crus I in working memory, planning and organization have been highlighted by functional imaging studies (Buckner, 2013). In addition, the role of DLPFC in executive function had been clearly established. This is consistent with our result that the FC between left Crus I and bilateral DLPFC correlated with execution. Previous fMRI also demonstrated the crossed cerebro-cerebellar projections, language is heavily right lateralized and visuospatial function left lateralized. Interestingly, in this study, we found similar lateralization in Crus I, as the FC of left Crus I connected with the execution and visuospatial function, and right Crus I connected with the memory and language.

Crossed cerebellar diaschisis (CCD) is the remote effect of supratentorial dysfunction in the unilateral hemisphere inducing contralateral cerebellar hypometabolism (Lin, 1997), which could explain the weakened FC between cerebellum and cerebrum. The mechanism of CCD include the involvement of cortico-ponto-cerebellar and the cerebello-thalamo-cortical circuits (Dum and Strick, 2003; Di Lorenzo et al., 2013). Using diffuse tension imaging (DTI), Sofia Toniolo et al. provide the evidence of the impairment of microstructural fiber integrity of cerebellum WM tracts in AD (Toniolo et al., 2020). However, whether the focal change in the cerebellum is a form of Wallerian degeneration or the result of accumulation of AD pathological substrates in the cerebellum itself is still unknown. In this study, we did not find morphometric difference in any of the observed cerebral cortical regions across the three groups, also implicating altered FC could be due to the dysfunction of neurotransmitter or network connection, instead of being secondary to the atrophy.

In this study, we did not find significant differences in volumes of any cerebellar lobular in AD or aMCI group from the data of our center, though the results based on ADNI database showed the GM volume loss in left Crus I/II. The smaller sample size and the younger age of our cohort might account for the discrepancy. Using the SUIT template for cerebellar VBM parcellation, which is the same method as our study, Sofia Toniolo et al. also reported a progression of cerebellar GM volume loss throughout a continuous spectrum from aMCI stage to AD stage (Toniolo et al., 2018).

To investigate the mechanism of cerebellar involvement in AD and aMCI is important for the diagnosis and treatment. Recent strategies of diagnosis and treatment for AD continuum are based on identification and quantification of the pathological biomarkers. Molecular neuroimaging study with selective radioligands, including Aβ and phosphorylated tau, is an important method for the quantification of these biomarkers (Jack et al., 2018). The most used parameter is the SUVR between a target region and a reference region (Fleisher et al., 2011). The selection of reference region directly affects the value of SUVR, which therefore is important for the diagnosis. The cerebellum has been the most widely used reference region in AD (Clark et al., 2011; Maass et al., 2017; Stern et al., 2019). However, if the cerebellum is involved in the pathogenesis of AD continuum, it may not be an optimal choice for the reference region. Furthermore, using the repetitive transcranial magnetic stimulation (rTMS) of cerebellum, Di Lorenzo et al. (2020) revealed the impairment of cerebellar-cortical plasticity by showing the long term potentiation (LTP) was impaired in AD patients. For the treatment, as the cerebellum is easily accessible with non-invasive stimulation tools, it may be used as a novel target for neuromodulation in AD in the future.

There are some limitations to this study. First, as a retrospective case-control study, the identification of the aMCI and AD groups was not based on pathological evidence, such as Aβ PET, which is still expensive for some patients in China. However, in this study, the prevalence of APOE ε4 carriers in AD and aMCI was 61.70 and 59.38%, respectively, which is similar to that of a previous study with large sample of Aβ biomarker positive individuals (66% in AD and 64% in MCI) (Mattsson et al., 2018). Second, though we included the aMCI group as the prodromal stage of AD, this was still a cross-sectional study. In the future, longitudinal studies are needed to investigate the dynamic changes in the cerebellum throughout disease progression.

## Conclusion

In conclusion, these findings suggest the functional changes of the cerebellum indicating the critical role cerebellum in the cognitive impairment in aMCI and AD. This was important because using the cerebellum as the reference region for ligand neuroimaging studies could bring the possible biased results.

## Data Availability Statement

The datasets presented in this study can be found in online repositories. The names of the repository/repositories and accession number(s) can be found below: http://dx.doi.org/10.17632/tc7xmjbmfw.3.

## Ethics Statement

The studies involving human participants were reviewed and approved by Research Ethics Committees of China-Japan Friendship Hospital. The patients/participants provided their written informed consent to participate in this study.

## Author Contributions

DP: conceptualization. ZZ, WS, and LW: methodology. ZZ, RZ, SZ, LW, and XD: formal analysis and investigation. ZZ: writing – original draft preparation. RZ and DP: writing – review and editing. DP: funding acquisition and supervision. All authors read and approved the final manuscript.

## Conflict of Interest

The authors declare that the research was conducted in the absence of any commercial or financial relationships that could be construed as a potential conflict of interest.

## Footnotes

1 http://www.diedrichsenlab.org/imaging/suit.htm

2 http://www.fil.ion.ucl.ac.uk/spm/

3 adni.loni.usc.edu

4 http://adni.loni.usc.edu/methods/documents/mri-protocols

5 http://www.adni-info.org/Scientists/AboutADNI.aspx
