# Corrigendum: Changes in Resting-State Functional Connectivity of Cerebellum in Amnestic Mild Cognitive Impairment and Alzheimer's Disease: A Case-Control Study

**DOI:** 10.3389/fnsys.2021.693951

**Published:** 2021-05-12

**Authors:** Zhi Zhou, Rui Zhu, Wen Shao, Shu-juan Zhang, Lei Wang, Xue-jiao Ding, Dan-tao Peng

**Affiliations:** ^1^Department of Neurology, China-Japan Friendship Hospital, Beijing, China; ^2^Department of Neurology, Beijing Geriatric Hospital, Beijing, China

**Keywords:** Alzheimer's disease, amnestic mild cognitive impairment, cerebellum, functional connectivity, resting state fMRI

In the published article, there was an error regarding the affiliation for Dan-tao Peng. Instead of “2,” the correct affiliation should be “1,” which is “Department of Neurology, China-Japan Friendship Hospital, Beijing, China.”

The authors apologize for this error and state that this does not change the scientific conclusions of the article in any way. The original article has been updated.

