# Supplementary material for: Changes in Resting-State Functional Connectivity of Cerebellum in Amnestic Mild Cognitive Impairment and Alzheimer’s Disease: A Case-Control Study
Source: Front Syst Neurosci. 2021 Mar 10;15:596221. doi: 10.3389/fnsys.2021.596221 (PMC8006280; doi:10.3389/fnsys.2021.596221)
Supplement: Supplementary file 1 [file Data_Sheet_1.docx]

Supplementary Material

# Supplementary Data

Cognitive tests

All subjects underwent a series of detailed cognitive tests—memory: Rey Auditory Verbal Learning Test (AVLT); language: 30-item version Boston Naming Test (BNT) ([1](#_ENREF_1)) and category fluency (animal) test; executive function: backward digit span (DS) and phonemic fluency (“Fa”)([2](#_ENREF_2)); attention: forward DS([2](#_ENREF_2)); visuospatial function: Wechsler Adult Intelligence Scale (WAIS)-III Block Design and clock-drawing test (CDT)([2](#_ENREF_2), [3](#_ENREF_3)).

# Supplementary Tables

2.1 Supplementary Table 1

**Supplementary Table 1. Neuropsychological data**

|  |  | **Normal Controls** | **Amnestic MCI** | **Alzheimer's disease** | ***p* ( ANOVA)** |
| --- | --- | --- | --- | --- | --- |
| N |  | 42 | 32 | 46 |  |
| AVLT, immediate recall | z-score | 0.10 ± 0.90 | -1.94 ± 0.51 | -2.73 ±1.13 | <0.001^1, 2, 3^ |
|  | Raw-score | 25.07 ± 4.42 | 15.00 ± 2.50 | 11.13 ± 5.58 |  |
| AVLT, delayed recall | z-score | 0.13 ± 0.71 | -2.26 ± 0.68 | -2.67 ± 0.69 | <0.001^1, 2^ |
|  | Raw-score | 9.71 ± 2.09 | 2.69 ± 2.15 | 1.48 ± 2.02 |  |
| Boston Naming Test | z-score | 0.22 ± 0.78 | -1.38 ± 1.53 | -1.94 ± 1.37 | <0.001^1,2^ |
|  | Raw-score | 23.69 ± 2.50 | 18.53 ± 4.94 | 16.72 ± 4.42 |  |
| Category fluency | z-score | 0.30 ± 1.07 | -1.20 ± 1.34 | -2.07 ± 1.21 | <0.001^1,2, 3^ |
|  | Raw-score | 15.50 ± 2.74 | 11.66 ± 3.45 | 9.42 ± 3.10 |  |
| Backward DS | z-score | -0.02 ± 1.06 | -0.73 ± 1.43 | -1.45 ± 1.15 | <0.001^1^ |
|  | Raw-score | 7.07 ± 1.98 | 5.75 ± 2.68 | 4.41 ± 2.15 |  |
| Phonemic fluency | z-score | -0.04 ± 1.01 | -0.48 ± 1.26 | -0.89 ± 1.41 | 0.020^1^ |
|  | Raw-score | 6.61 ± 1.92 | 5.78 ± 2.39 | 5.00 ± 2.68 |  |
| Forward DS | z-score | -0.07 ± 0.95 | -0.27 ± 1.30 | -0.63 ± 1.03 | 0.050 |
|  | Raw-score | 9.81 ± 1.82 | 9.41 ± 2.51 | 8.72 ± 1.99 |  |
| CDT | z-score | -0.03 ± 0.99 | -0.86 ± 1.50 | -4.12 ± 3.98 | <0.001^1, 3^ |
|  | Raw-score | 28.21 ± 2.17 | 26.41 ± 3.27 | 19.30 ± 8.69 |  |
| Block Design | z-score | 30.67 ± 8.11 | -0.83 ± 1.02 | 14.80 ± 9.98 | <0.001^1, 3^ |
|  | Raw-score | -0.18 ± 1.13 | 26.00 ±7.30 | -2.40 ± 1.40 |  |

Abbreviations: AVLT, auditory verbal learning test; Forward DS, forward digit span; Backward DS, backward digit span. CDT, clock drawing test.

1. Post-hoc analysis showed significant group differences between NC and AD. 2. Post-hoc analysis showed significant group differences between NC and aMCI. 3. Post-hoc analysis showed significant group differences between aMCI and AD.

2.2 Supplementary Table 2

| **Supplementary Table 2**  **Pearson Correlation between cognition and cerebellar FC in aMCI and AD group** | | | | | | | | | |
| --- | --- | --- | --- | --- | --- | --- | --- | --- | --- |
| **z score** | | | | **Composite** | **Memory** | **Language** | **Executive** | **Attention** | **Visuospatial** |
| **Left VIIb** | **Right BA 18, 19** | **aMCI** | ***r*** | -0.031 | 0.031 | -0.189 | 0.004 | -0.171 | 0.296 |
|  |  |  | ***p*** | 0.864 | 0.866 | 0.299 | 0.982 | 0.349 | 0.100 |
|  |  | **AD** | ***r*** | 0.177 | -0.034 | 0.026 | 0.335 | -0.021 | 0.130 |
|  |  |  | ***p*** | 0.238 | 0.824 | 0.862 | 0.023 | 0.891 | 0.395 |
| **Left IX** | **Left**  **BA 18, 19** | **aMCI** | ***r*** | 0.071 | 0.045 | -0.100 | 0.091 | 0.039 | 0.172 |
|  |  |  | ***p*** | 0.701 | 0.809 | 0.587 | 0.620 | 0.834 | 0.346 |
|  |  | **AD** | ***r*** | 0.002 | 0.216 | 0.155 | -0.197 | 0.183 | -0.168 |
|  |  |  | ***p*** | 0.990 | 0.149 | 0.304 | 0.190 | 0.228 | 0.270 |
|  | **Right**  **BA 18,19** | **aMCI** | ***r*** | 0.041 | 0.034 | 0.098 | 0.040 | -0.316 | 0.323 |
|  |  |  | ***p*** | 0.823 | 0.853 | 0.595 | 0.827 | 0.078 | 0.071 |
|  |  | **AD** | ***r*** | 0.162 | 0.363 | 0.144 | 0.039 | 0.152 | -0.020 |
|  |  |  | ***p*** | 0.283 | 0.013 | 0.340 | 0.797 | 0.319 | 0.899 |
| **Left Crus I** | **Left**  **BA 9,32** | **aMCI** | ***r*** | -0.062 | -0.110 | -0.042 | 0.054 | -0.296 | 0.174 |
|  |  |  | ***p*** | 0.736 | 0.548 | 0.821 | 0.771 | 0.100 | 0.342 |
|  |  | **AD** | ***r*** | 0.186 | 0.050 | 0.051 | 0.204 | 0.082 | 0.185 |
|  |  |  | ***p*** | 0.216 | 0.743 | 0.735 | 0.174 | 0.592 | 0.224 |
|  | **Right**  **BA 45, 46** | **aMCI** | ***r*** | 0.127 | 0.061 | 0.018 | 0.173 | -0.239 | 0.440 |
|  |  |  | ***p*** | 0.487 | 0.740 | 0.923 | 0.344 | 0.187 | 0.012 |
|  |  | **AD** | ***r*** | 0.216 | 0.048 | 0.225 | 0.166 | 0.121 | 0.109 |
|  |  |  | ***p*** | 0.149 | 0.749 | 0.132 | 0.270 | 0.428 | 0.477 |
| **Right Crus I** | **Left**  **BA 19,37** | **aMCI** | ***r*** | -0.138 | 0.300 | 0.016 | -0.012 | -0.227 | -0.310 |
|  |  |  | ***p*** | 0.452 | 0.095 | 0.931 | 0.949 | 0.211 | 0.084 |
|  |  | **AD** | ***r*** | 0.330 | 0.334 | 0.321 | 0.195 | 0.229 | 0.059 |
|  |  |  | ***p*** | 0.025 | 0.023 | 0.030 | 0.194 | 0.131 | 0.701 |
|  | **Right**  **BA 21, 37** | **aMCI** | ***r*** | -0.106 | 0.047 | -0.258 | 0.055 | -0.061 | -0.057 |
|  |  |  | ***p*** | 0.564 | 0.797 | 0.154 | 0.765 | 0.738 | 0.757 |
|  |  | **AD** | ***r*** | 0.259 | 0.259 | .312* | 0.032 | 0.245 | 0.020 |
|  |  |  | ***p*** | 0.082 | 0.082 | 0.035 | 0.834 | 0.105 | 0.895 |
| Abbreviations: aMCI: amnestic mild cognitive impairment; naMCI: non-amnestic mild cognitive impairment.  Bold and *for *p*<0.05. | | | | | | | | | |

## 2.3 Supplementary Table 3

| **Table 3. Significant different clusters among the AD, aMCI and NC groups from ADNI database** | | | | | | | |
| --- | --- | --- | --- | --- | --- | --- | --- |
|  | Cluster voxels | Brain regions | MNI coordinate | | | Maximum *F* |  |
|  |  |  | x | y | z |  |  |
| ANOVA | 49 | Left Crus I  Left Crus II | -22 | -84 | -35 | 29.074 |  |
| Post-hoc analysis | *NC > aMCI: *p* < 0.001; *NC > AD：*p*< 0.0001; aMCI > AD: *p* = 0.059 | | | | | | |
| NC: normal control；aMCI：amnestic mild cognitive impairment；AD: Alzheimer’s disease.  * The difference between NC and AD, NC and aMCI survived at Bonferroni's correction in the post-hoc analyses | | | | | | | |

## 2.4 Supplementary Table 4

| **Supplementary Table 4.** **Brain regions showing significant differences during one-way ANOVA on z value of functional connectivity maps of NC, aMCI, and AD groups from ADNI database** | | | | | | | | | |
| --- | --- | --- | --- | --- | --- | --- | --- | --- | --- |
| **Seed** | **Cluster**  **Voxels** | **Brain regions** | **Laterality** |  | **MNI coordinate** | | | **Maximum *F*** | **Post-hoc** |
|  |  |  |  |  | **x** | **y** | **z** |  |  |
| Left IX | 38 | PHG | Left |  | -24 | -24 | 21 | 16.00 | *NC > aMCI: *p* < 0.001;  *NC > AD：*p*< 0.001;  aMCI > AD: *p* > 0.999 |
| Right X | 37 | ITG, FG | Left |  | -39 | -36 | 24 | 20.51 | NC > aMCI: *p* =0.441;  *NC > AD：*p*< 0.001;  *aMCI > AD: *p* < 0.001 |
|  | 21 | PHG | Left |  | -18 | -30 | -9 | 21.64 | NC > aMCI: *p* = 0.646;  *NC > AD：*p*< 0.001;  *aMCI > AD: *p*< 0.001 |
|  | 42 | MCC | Bilateral |  | -3 | -36 | 48 | 21.65 | NC > aMCI: *p* = 0.272;  *NC > AD：*p*< 0.001;  *aMCI > AD: *p*< 0.001 |
| Left Crus I | 41 | SFG, MFG, DLPFC | Left |  | -9 | 54 | 27 | 18.63 | *NC > aMCI: *p* < 0.001;  *NC > AD：*p*< 0.001;  aMCI > AD: *p* = 0.041 |
|  | 28 | SFG | Right |  | 6 | 51 | 33 | 16.90 | *NC > aMCI: *p* < 0.001;  *NC > AD：*p*< 0.001;  aMCI > AD: *p* = 0.241 |
|  | 32 | Pcu,Cu | Left |  | -6 | -60 | 45 | 16.34 | *NC > aMCI: *p* < 0.001;  *NC > AD：*p*< 0.001;  aMCI > AD: *p* = 0.039 |
| Right Crus I | 37 | Pcu,Cu | Right |  | 12 | -78 | 30 | 19.17 | *NC > aMCI: *p* = 0.016;  *NC > AD：*p*< 0.001;  aMCI > AD: *p* = 0.049 |
|  | 70 | Parietal lobe, Pcu,Cu | Left |  | -6 | -60 | 39 | 19.35 | *NC > aMCI: *p* = 0.016;  *NC > AD：*p*< 0.001;  *aMCI > AD: *p* = 0.004 |
| Right Crus II | 21 | MCC | Bilateral |  | -3 | -33 | 48 | 27.02 | *NC > aMCI: *p* = 0.001;  *NC > AD：*p*< 0.001;  *aMCI > AD: *p*< 0.001 |
| Abbreviations: BA: Brodamann area. PCu: precuneus; Cu: cuneus; DLPFC: dorsolateral prefrontal cortex; IFG: inferior frontal gyrus; SFG: Superior Frontal Gyrus; MFG: middle frontal gyrus; MCC: middle cingulate cortex; MTG: middle temporal gyrus; Parahippocampa Gyrus: PHG. Fusiform Gyrus: FG. Inferior temporal gyrus: ITG  * The difference between NC and AD, NC and aMCI survived at Bonferroni's correction in the post-hoc analyses | | | | | | | | | |

## 3.1 Supplementary Figures


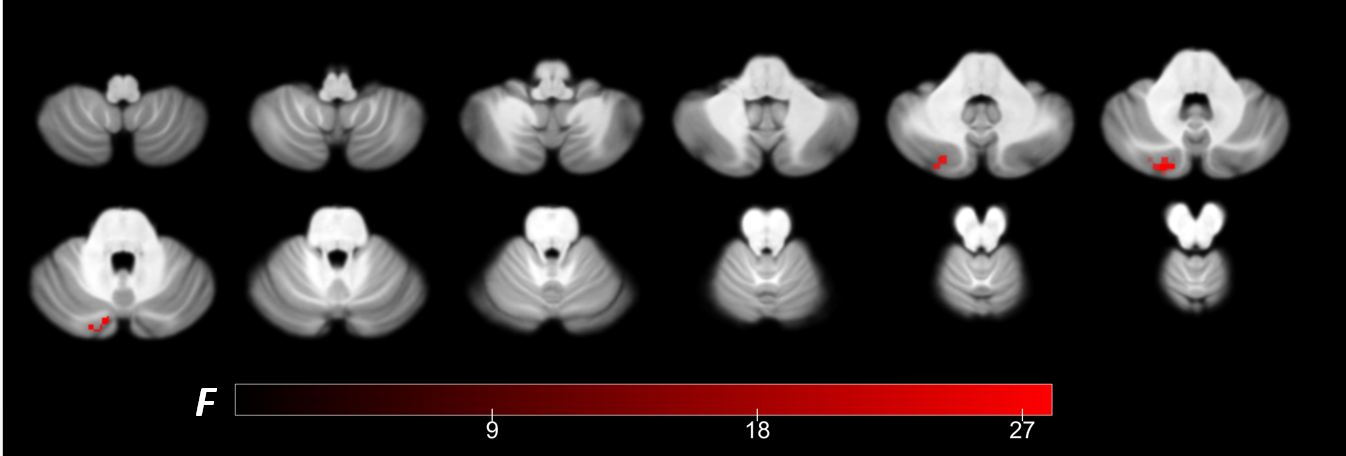


**Supplementary Figure 1.** The significant different clusters in cerebellar volume among the AD, aMCI, and NC groups. The significant areas included the left Crus I/II lobe. Color bar represents *F* values. (Neurological view: right of image is right of brain).


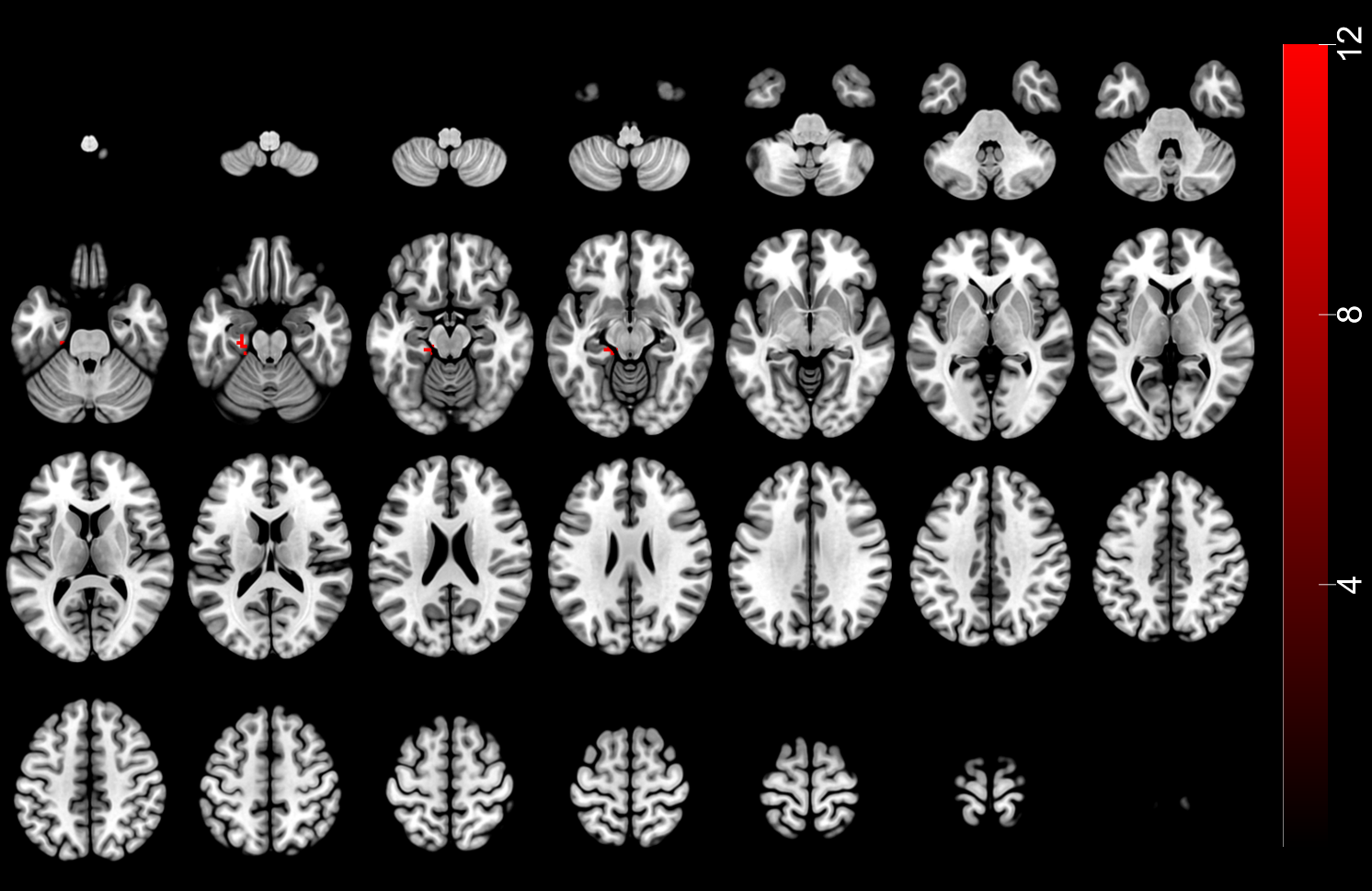


**Supplementary Figure 2.** The significant different cluster of the FC of left IX lobe among AD, aMCI and NC group. Color bar represents *F* values. (Neurological view: right of image is right of brain).

**
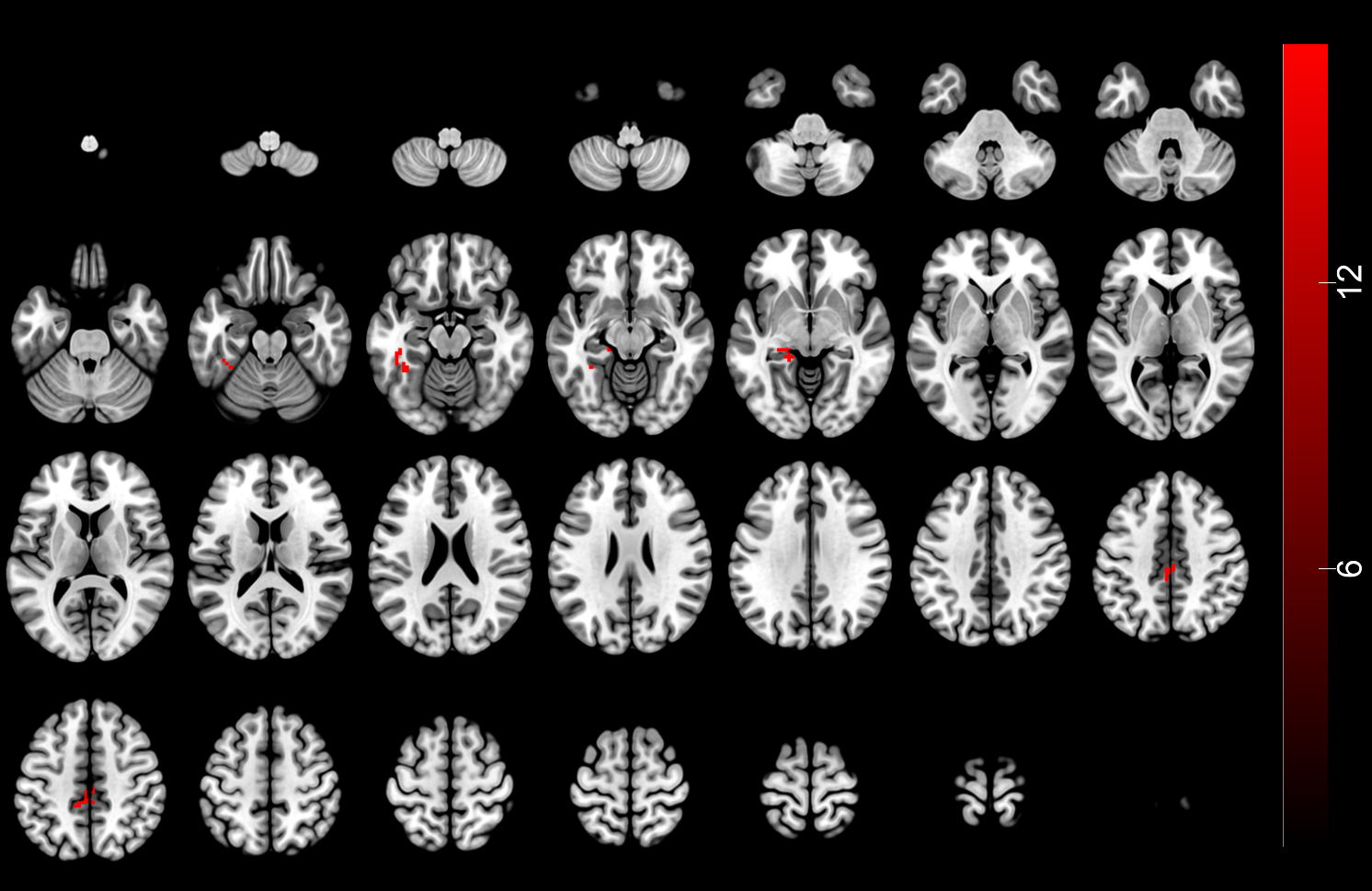
**

**Supplementary Figure 3.** The significant different cluster of the FC of Right X lobe among AD, aMCI and NC group. Color bar represents *F* values. (Neurological view: right of image is right of brain).

**
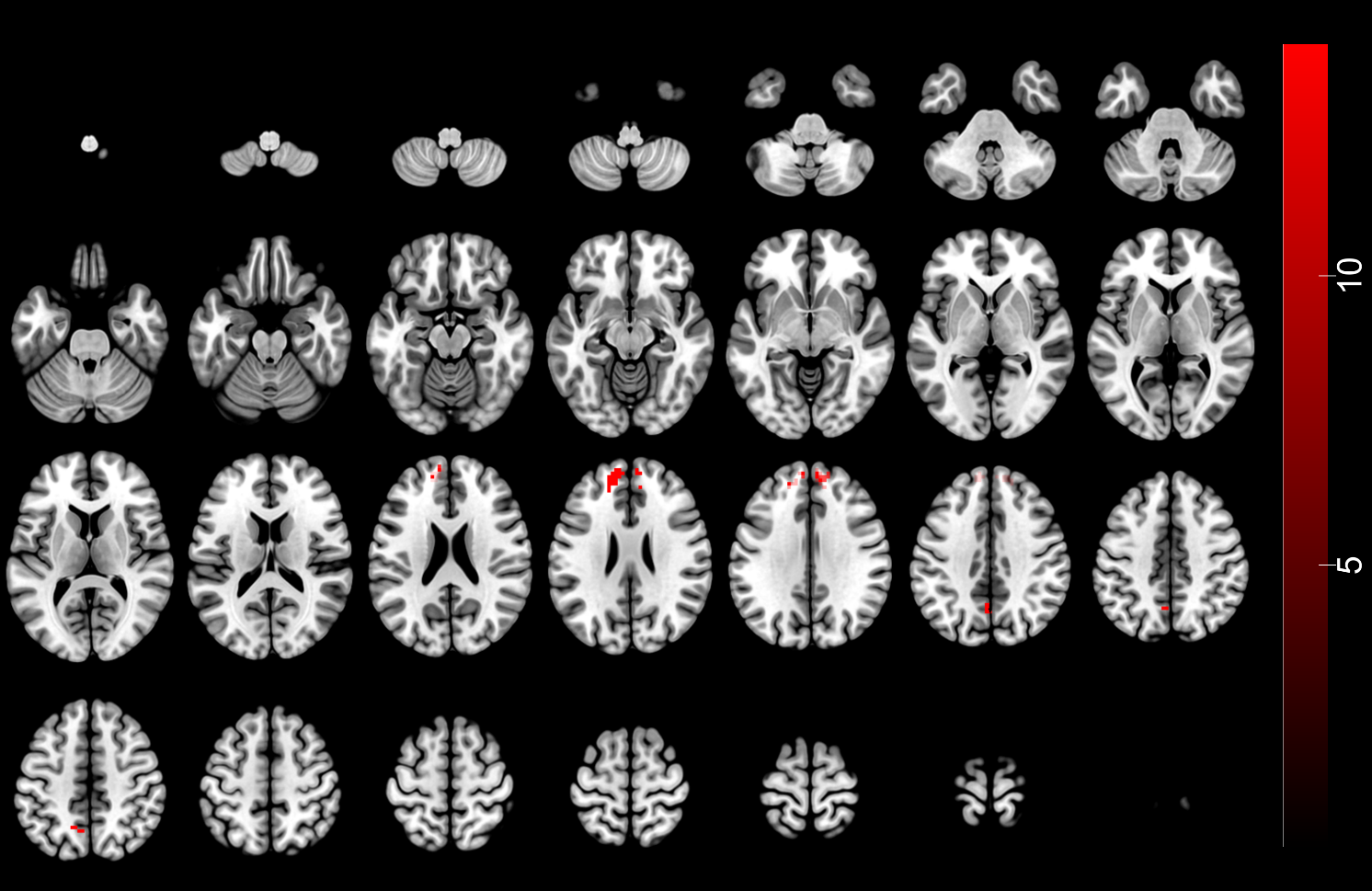
 Supplementary Figure 4.** The significant different cluster of the FC of left Crus I among AD, aMCI and NC group. Color bar represents *F* values. (Neurological view: right of image is right of brain).

**
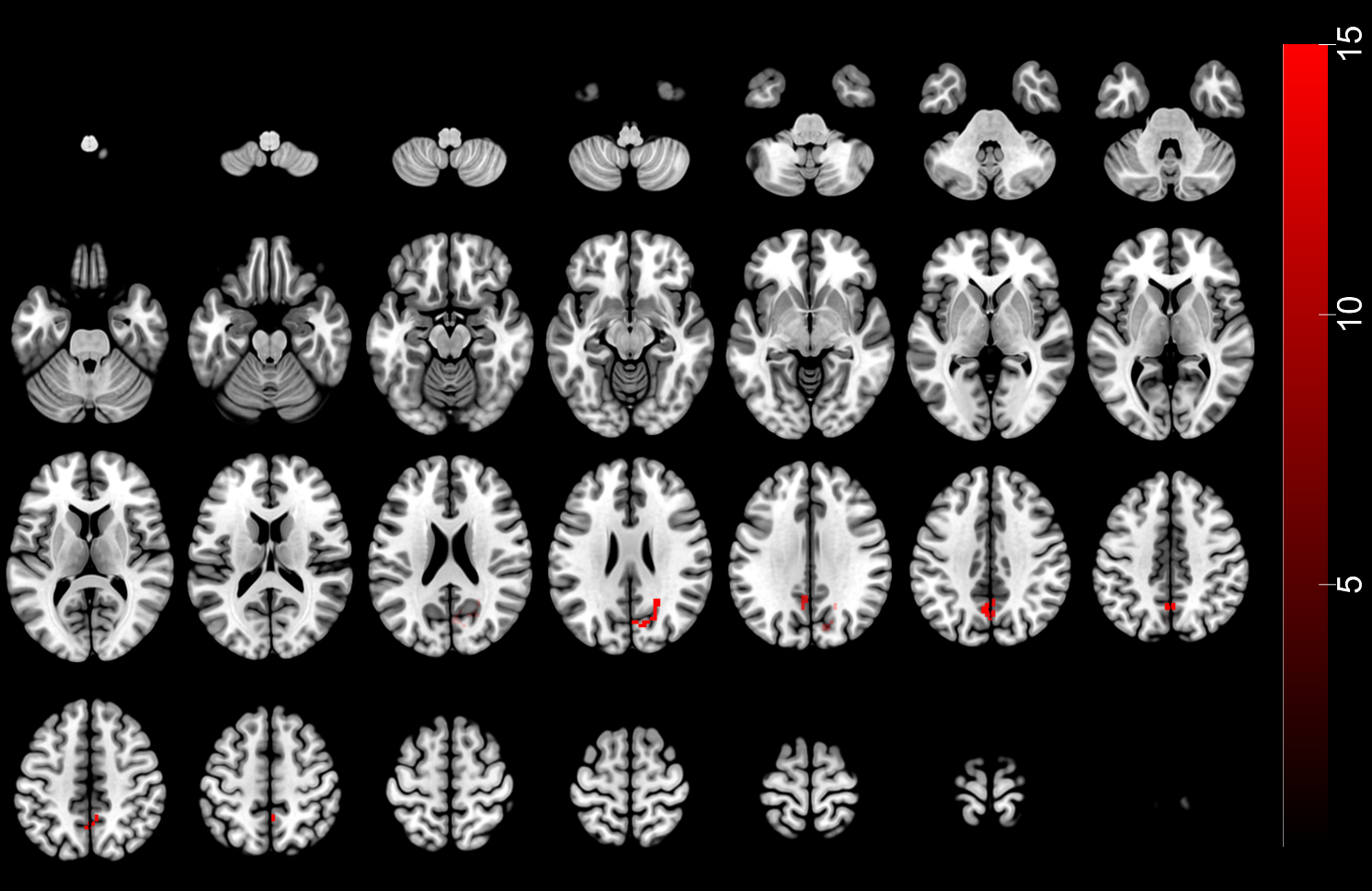
**

**Supplementary Figure 5.** The significant different cluster of the FC of right Crus I among AD, aMCI and NC group. Color bar represents *F* values. (Neurological view: right of image is right of brain).

**
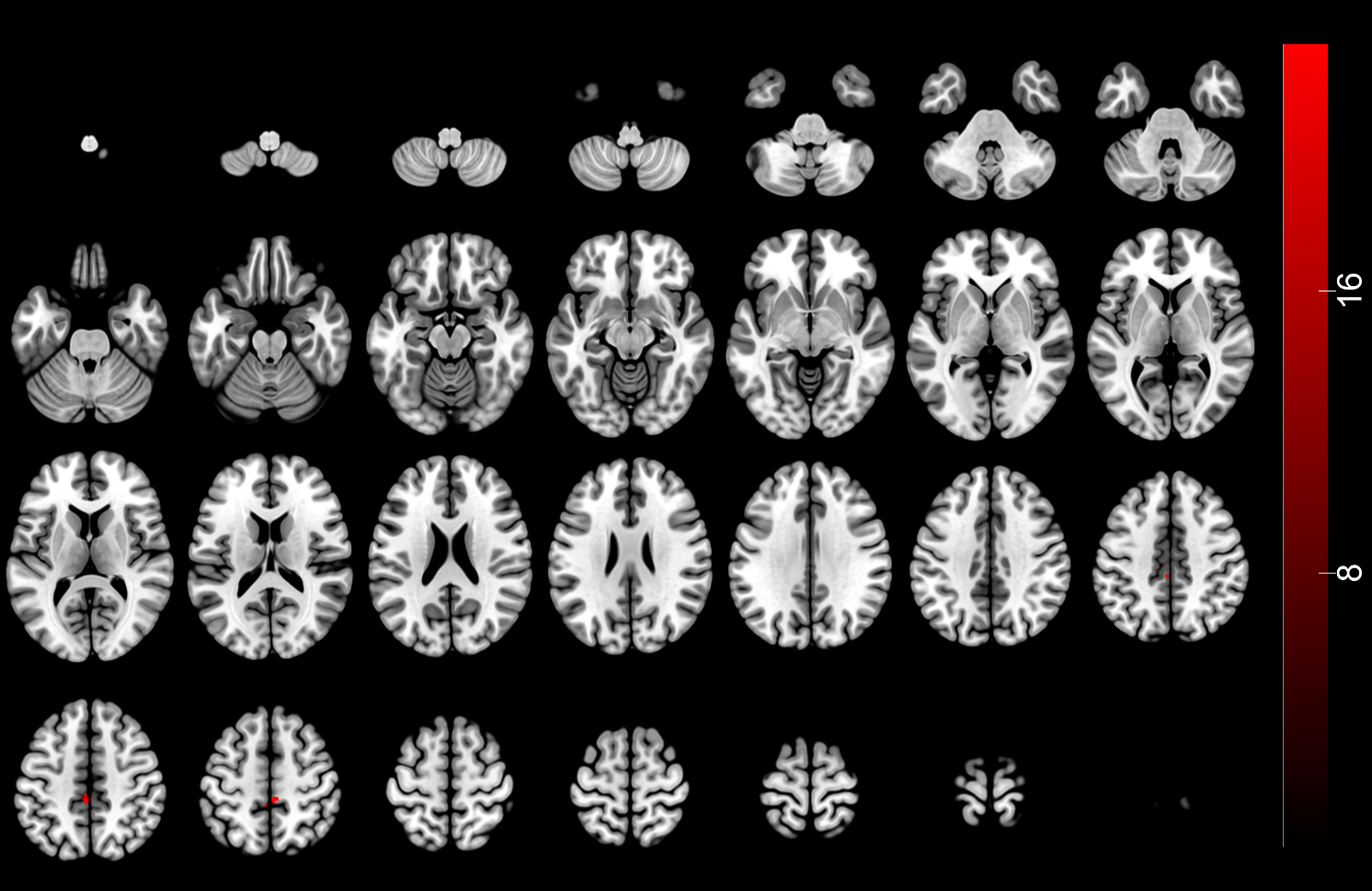
**

**Supplementary Figure 6.** The significant different cluster of the FC of right Crus II among AD, aMCI and NC group. Color bar represents *F* values. (Neurological view: right of image is right of brain).

**References**

1. Williams BW, Mack W, Henderson VW. Boston Naming Test in Alzheimer's disease. *Neuropsychologia* (1989) 27(8):1073-9. PubMed PMID: 2797414.

2. Wechsler D. Wechsler Adult Intelligence Scale—Third Edition (WAIS–III). 1997. *San Antonio: The Psychological Corporation*.

3. Agrell B, Dehlin O. The clock-drawing test. *Age and ageing* (1998) 27(3):399-403.
